# Sex-specific selection for MHC variability in Alpine chamois

**DOI:** 10.1186/1471-2148-12-20

**Published:** 2012-02-15

**Authors:** Helmut Schaschl, Franz Suchentrunk, David L Morris, Hichem Ben Slimen, Steve Smith, Walter Arnold

**Affiliations:** 1 Department for Integrative Biology and Evolution, Research Institute of Wildlife Ecology, University of Veterinary Medicine Vienna, Savoyenstrasse 1, 1160 Vienna, Austria; 2 Divisions of Genetics and Molecular Medicine and Immunology, Infection and Inflammatory Disease, King's College London, London WC2R 2LS, UK; 3 Institut Supérieur de Biotechnologie de Béja, Habib Bourguiba Street, Beja 9000, P.O.Box 382, Tunisia; 4 School of Biological Sciences, Flinders University in Adelaide, GPO Box 2100, Adelaide, SA 5001, Australia

**Keywords:** MHC, Sex-specific selection, Heterozygosity advantage, Alpine chamois

## Abstract

**Background:**

In mammals, males typically have shorter lives than females. This difference is thought to be due to behavioural traits which enhance competitive abilities, and hence male reproductive success, but impair survival. Furthermore, in many species males usually show higher parasite burden than females. Consequently, the intensity of selection for genetic factors which reduce susceptibility to pathogens may differ between sexes. High variability at the major histocompatibility complex (MHC) genes is believed to be advantageous for detecting and combating the range of infectious agents present in the environment. Increased heterozygosity at these immune genes is expected to be important for individual longevity. However, whether males in natural populations benefit more from MHC heterozygosity than females has rarely been investigated. We investigated this question in a long-term study of free-living Alpine chamois (*Rupicapra rupicapra*), a polygynous mountain ungulate.

**Results:**

Here we show that male chamois survive significantly (*P *= 0.022) longer if heterozygous at the MHC class II *DRB *locus, whereas females do not. Improved survival of males was not a result of heterozygote advantage *per se*, as background heterozygosity (estimated across twelve microsatellite loci) did not change significantly with age. Furthermore, reproductively active males depleted their body fat reserves earlier than females leading to significantly impaired survival rates in this sex (*P *< 0.008). This sex-difference was even more pronounced in areas affected by scabies, a severe parasitosis, as reproductively active males were less likely to survive than females. However, we did not find evidence for a survival advantage associated with specific MHC alleles in areas affected by scabies.

**Conclusions:**

Increased MHC class II *DRB *heterozygosity with age in males, suggests that MHC heterozygous males survive longer than homozygotes. Reproductively active males appear to be less likely to survive than females most likely because of the energetic challenge of the winter rut, accompanied by earlier depletion of their body fat stores, and a generally higher parasite burden. This scenario renders the MHC-mediated immune response more important for males than for females, which implies a relatively stronger selection pressure on MHC genes in males than in females.

## Background

Longevity depends on effective immune defence against the multitude of pathogens that an individual encounters over its lifetime. Increased heterozygosity, or specific alleles at immune loci, are therefore expected to be important for individual survival. Key molecules in directing important parts of the adaptive immune response are encoded by the MHC class I and class II genes. These highly polymorphic MHC genes express molecules that present antigenic peptides on the cell surface to T-cells, thereby initiating the T-cell mediated immune responses in vertebrates. MHC molecules also play a key role in directing and shaping the T-cell receptor repertoire during T-cell maturation (i.e. T-cell restriction). Heterozygosity at MHC loci may therefore enhance resistance to infectious diseases by binding and presenting a wider range of antigens to T-cells on the one hand [1], while on the other, generating a more diverse T-cell receptor repertoire during T-cell maturation [2-4]. As a result, MHC heterozygous individuals are thought to have enhanced immunity to environmental pathogens (i.e., heterozygosity advantage) (reviewed in [5]). In fact, there is substantial evidence that pathogen-driven selection enhances MHC diversity. MHC heterozygosity has been associated with a more effective clearance rate of infection [4,6-8], reduced parasite load and spectrum [9,10], higher reproductive ability [11,12], and higher survival [4,13-15]. In contrast, other studies have found allele-specific associations with parasite load and no benefits for heterozygosity *per se *[16-20]. An efficient immune response will favor the spread of new, less detectable strains of a pathogen. As a result, previously rare, and presumably less effective, MHC variants may combat these new strains better than previously selected alleles (i.e., negative frequency-dependent selection; reviewed in [21]). This selection scenario is expected to cause cyclic selection, driving rare alleles to relatively high frequency before they are selected against [22].

In mammals, including humans, males typically have shorter lives than females [23,24]. In particular, males of polygynous mammals show higher rates of mortality than females. This difference is thought to be due to high energy expenditure while competing for access to females that impairs subsequent survival [23,25]. In particular, the immune function may be impaired in males by activities associated with reproductive effort. Indeed, in many species, males show higher parasite burden than females [26]. Consequently, selective pressures on immune genes, such as MHC genes, may differ between males and females. However, whether males in natural populations benefit more from MHC heterozygosity than females has rarely been studied.

We investigated this question in a long-term study of free-living Alpine chamois (*Rupicapra rupicapra*), a highly polygynous mountain ungulate, which belongs to the Bovidae family and Caprinae subfamily. This species preferentially inhabits alpine pastures and rocky areas in diverse mountain regions of Europe and the Middle East. Chamois rut at the beginning of winter and reproductively active males are believed to have high energy expenditure which depletes body fat stores that are pivotal for surviving the harsh and long alpine winter [27,28]. The life expectancy in free-living chamois is about 16-20 years [29]. Males and females show, in large part, overlapping survival curves but male survival decreases appreciably at the age of 11 years [29]. Similarly, in the closely related Pyrenean chamois (*Rupicapra pyrenaica*) survival was found to be lower in males than females and this gender difference increased also with age [24] (but see [30]).

The most severe parasitosis affecting Alpine chamois is sarcoptic mange or scabies. This highly contagious disease, caused by a submacroscopic ectoparasite (mites, *Sarcoptes rupicaprae*) that burrows into the skin causing intense itching, hyperkeratosis, severe skin lesions, subsequent bacterial infections and eventually death [31,32]. This disease is usually transmitted directly by skin-to-skin contact with individuals already infested with the mites. Scabies epidemics occur in more or less regular waves across large parts of the Eastern Alps (e.g., in Austria and Italy) and can play an important role in the dynamics of chamois populations with local mortality rates of up to 80% [33,34]. Mortalities occur mainly at the end of the harsh alpine winter when the animals, and hence their immune defences, are weakened after months of cold temperatures and restricted food supplies [35]. The immune response to sarcoptic mange infestation is complex and still poorly understood, however scabies infection appears to initiate a specific and strong antibody response, indicating the activation of the adaptive immune system [36,37]. The immune response of chamois to this parasite infection may also vary in intensity, depending on the individual MHC variability. Chamois expresses a single polymorphic MHC class II *DRB *gene [38,39]. Codon-based tests for selection showed that the peptide-binding region (encoded by the exon 2) of this gene contains a significantly higher rate of non-synonymous than synonymous substitutions (d_N_/d_S_), indicating strong positive selection for diversity on a large evolutionary time scale [40,41].

**Figure 1:**
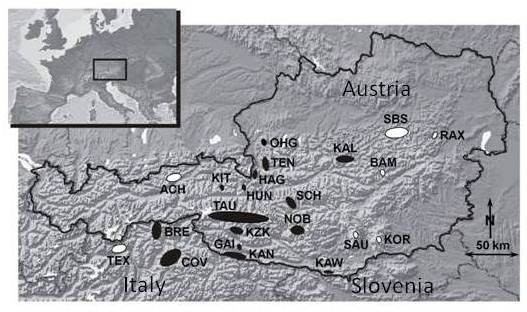
**Geographic location of the 22 chamois sampling sites in the Eastern Alps of Austria and the province of South Tyrol (Alto Adige) in Italy**. Size and shape of the spots represent rough estimates of the sampled areas. Black spots indicate the 15 localities where scabies outbreaks occurred during the study period. Abbreviations: ACH Achenkirch, BAM District Bruck an der Mur, BRE District Brenner (Italy), COV Corvara (Italy), GAI Gailtaler Alpen, HAG Hagengebirge, HUN Hundstein, KAL Kalkalpen, KAN Karnische Alpen, KAW Karawanken, KIT Kitzbüheler Alpen, KOR Koralm, KZK Kreuzeckgruppe, NOB Nockberge, OHG Osterhorngruppe, RAX Rax, SAU Saualm, SBS District Scheibbs, SCH Schladminger Tauern, TAU Hohe Tauern, TEN Tennengebirge, TEX Texelgruppe (Italy).

In the present study, we examined whether individual survival of males and females differed in chamois populations that had been exposed to scabies epidemics historically and during the timeframe of our study, or were thought to be never affected by scabies (Figure 1), and whether mortality was associated with variability at the MHC class II *DRB *locus. To control for potentially confounding differences in overall genetic variability, we additionally screened genetic variation at 12 microsatellite loci. We further examined energetic costs of rutting in this species to investigate whether these costs could influence survival rates in males and females.

## Results

### MHC and microsatellite variability

**Figure 2:**
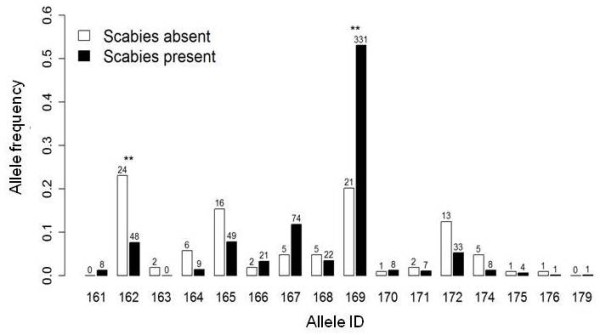
**MHC class II *DRB *allele frequencies in chamois for regions where scabies has occurred during the study period (black), or had never been reported (white)**. Numbers above bars indicate the total number of alleles found; ** indicates a significant difference in MHC allele frequencies with *P *< 0.01 (Fisher's exact test, adjusted for multiple comparisons).

We detected 16 MHC class II *DRB *alleles that varied considerably in frequency (Figure 2). The corresponding nucleotide sequences (GenBank accession numbers) can be found in Additional file 1: Table S1. The mean expected heterozygosity (H_E_) for the MHC class II *DRB *locus was 0.73 (standard deviation [s.d.] = 0.13) and for the 12 analyzed microsatellite loci 0.69 (s.d. = 0.14). We did not find significant linkage between the MHC locus and the microsatellite loci (*P *> 0.05 after Bonferroni correction). Genetic diversity at the MHC locus was significantly (F_1.19 _= 15.22, *P *= 0.001) lower for chamois populations affected by scabies (H_E _= 0.69) than for those unaffected by scabies (H_E _= 0.84). However, lower genetic diversity in populations where scabies had occurred was unlikely to be the result of merely genetic drift, as indicated by the much higher *F*_ST _value of the MHC locus (overall *F*_ST _= 0.063, *P *< 0.05) compared to that of the twelve microsatellite loci (overall *F*_ST _= 0.019, *P *< 0.05). The identified MHC alleles differed profoundly in frequencies between chamois populations exposed to scabies and those where scabies epidemics have never been reported (Figure 2). Particularly, the frequency of the most dominant allele (169, freq = 0.5) was about two times higher in scabies affected populations, which may explain why heterozygosity at the MHC locus was lower in these populations (partial effect, *z *= -2.58, *p *= 0.010). Except for allele 169, only allele 162 differed significantly in frequency between populations affected by scabies or not (*P *< 0.01 after Benjamini-Hochberg adjustment for multiple comparisons with Fisher's exact test; all other tests *P *> 0.05, Figure 2).

### Selection at the MHC class II DRB

**Figure 3:**
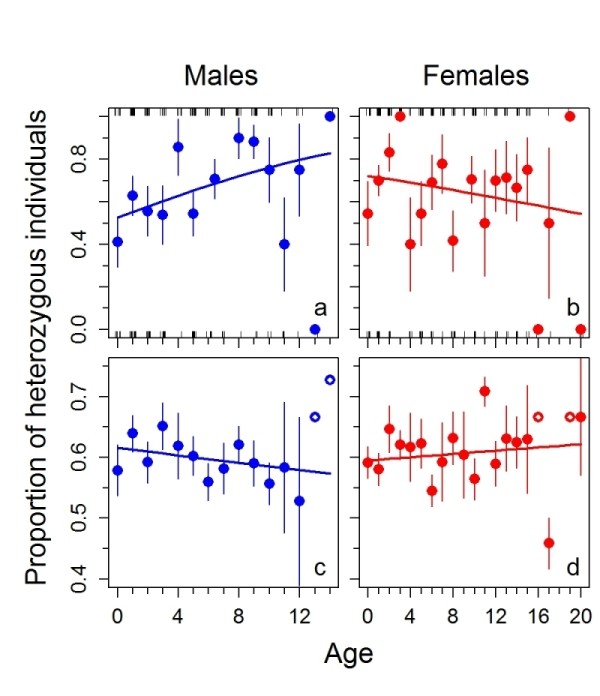
**Sex-specific changes of heterozygosity with age in chamois**. **a-b **Proportions of males (blue) and females (red) within age classes being heterozygous at the MHC class II *DRB *locus (± standard errors of binomial proportions); thick lines are predictions from logistic regressions; bars along the x-axes (slightly spaced) visualize the number of hetero- (above) and homozygous individuals (below) in each age class. **c-d **Average heterozygosity of males (blue) and females (red) within age classes at 12 microsatellite loci (± s.e., thick lines are predictions from linear regressions; sample sizes of one are indicated by symbols with white dots). See text for statistics.

To examine for age-related changes in frequency of MHC class II *DRB *genotypes, indicating an association with survival, we regressed in a generalized linear model (GLM) the binomially coded MHC genotypes (hetero- *vs*. homozygous) against age (in years). We further included sex in the model to test for potential differences between males and females, and average individual heterozygosity at the microsatellite loci to control for background genetic variability. The most parsimonious model found with our model averaging approach (see Methods) contained only one significant term, the interaction of sex and age (z = 2.38, *P *= 0.018; relative importance of the predictors remaining in the final model: sex = 0.60, age = 0.54, sex * age = 0.41, average microsatellite heterozygosity = 0.29, average microsatellite heterozygosity * sex = 0.04, average microsatellite heterozygosity * age = 0.03). Separate GLMs for both sexes revealed an increase of MHC heterozygosity with age in males (deviance = 5.31, *P *= 0.022), but not in females (deviance = 1.78, *P *= 0.185; Figure 3a,b). The median age at death for MHC homozygous males was 3 years and for MHC heterozygous males 6 years. Average heterozygosity at the microsatellite loci did not change significantly with age in either sex (males: *F*_(1,168) _= 0.91, *P *= 0.342; females: *F*_(1,188) _= 0.44, *P *= 0.509; Figure 3c,d). However, specific MHC alleles, and not heterozygosity *per se*, could be responsible for enhanced immune defence. We therefore tested each identified MHC allele for an increase in frequency with age, but found no statistical evidence for an association of certain MHC alleles and increased age in either males or females.

### Energetic cost of rutting and survival rate

**Figure 4:**
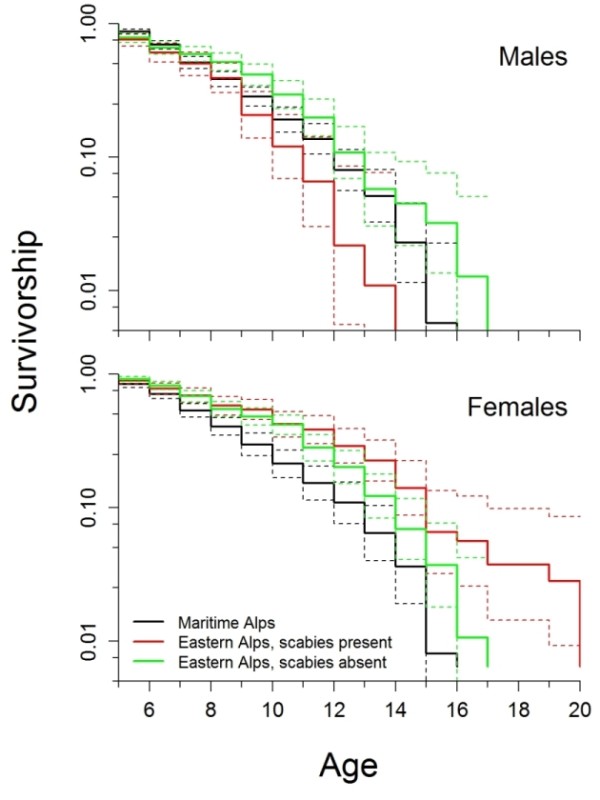
**Analysis of female and male survival in chamois**. Cox proportional survival hazards (thick lines) and 95% confidence intervals (thin lines) of reproductively active males and females in populations of the Eastern Alps affected by scabies (red), or where scabies was never reported (green). Reproductive activity was assumed for animals older than four years, the age when males first engage in rutting [42]. Black lines indicate survivorships of chamois in the Maritime Alps Regional Park in Italy, an area where scabies never occurred and hunting is banned; these survivorships were estimated from carcasses found in the natural habitat and aged by [29]. Survivorship curves are plotted on a logarithmic scale representing the proportion of individuals surviving to a certain age.

**Figure 5:**
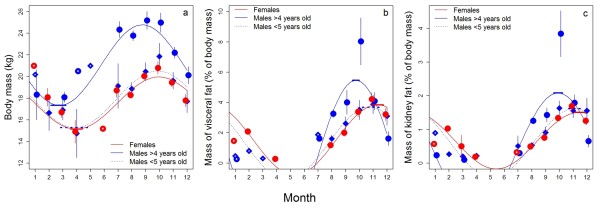
**Seasonal changes of body mass and fat reserves in chamois**. **a **Seasonal changes of the eviscerated body mass of males (blue) and females (red), **b **of relative visceral fat mass, and **c **of relative kidney fat mass. Among males, we discriminated between reproductively active males older than four years [42] (circles, solid lines), and younger animals (diamonds, dashed line). Plotted are monthly means ± standard errors with sample sizes of one indicated by symbols with white dots. Sinoidal curves are the seasonal mass changes predicted from cosinor analysis (all amplitudes differ from 0 with *P *< 0.001). Horizontal thick lines indicate the 95% confidence interval of the estimated date of the annual nadir (**a**), or corresponding peaks (**b,c**), respectively.

Firstly, we checked, with Cox's proportional hazards analysis, whether the age structure in our sample of hunted chamois reflected natural mortality by comparing it with 508 male and 359 female age-known carcasses found during a study in the Western Alps (Maritime Alps Regional Park) in Italy [29] (Figure 4). Hunting is banned in this park and scabies has never occurred there. Thus, all of these individuals had died naturally but not from scabies. Survivorship curves for males older than 4 years from this sample did not differ significantly from our sample of populations affected by scabies (*P_ad j _*= 0.299), nor from that for populations where scabies had presumably never occurred (*P_adj _*= 0.232). In contrast, survival of females older than 4 years from the Maritime Alps population was lower than that of females in our sample (difference to females from populations affected by scabies, *P_adj _*< 0.001; to females from populations where scabies was never reported, *P_adj _*= 0.001) (Figure 4). Secondly, modeling the annual course of body mass and body fat reserves we found nadirs at the end of winter (Figure 5), with reproductively active males (older than 4 years [42]) reaching lowest body mass (32% below the autumn peak, 95% confidence interval (CI) = 21.2-43.2) about six weeks before females (annual nadir 25% below the autumn peak, CI = 15.7 - 34.8; Figure 5). This was most likely due to the high energy expenses of these males during the rut in early winter, as younger males, which typically are not engaged in rutting, had seasonal body mass and body reserve trajectories more similar to females (Figure 5). As a result, survival of reproductively active males was significantly lower, but only in populations affected by scabies (Figure 5, differences in Cox's proportional hazards, adjusted for multiple comparisons of the six survivorship curves shown [Tukey's honest significant difference]: compared to females from populations affected by scabies, *P_adj _*< 0.001; to males from populations without scabies, *P_adj _*= 0.008; to females from populations without scabies *P_adj _*< 0.001). In contrast, survival of females did not differ significantly according to presence or absence of scabies (*P_adj _*= 0.301), and was similar to that of males in populations thought to have never been affected by scabies (*P_adj _*= 0.251).

## Discussion

Both the survival analysis and genetic data from our study demonstrate higher mortality for reproductively active males compared to females and younger males. We showed that heterozygosity at the MHC class II *DRB *locus increased with age in males, but not in females. This difference between the sexes was significant and independent from background genetic variability (i.e., heterozygosity at 12 microsatellite loci) (Figure 3). The mutational processes for MHC and microsatellites are different and therefore allelic diversity of these genetic markers cannot be compared directly. However, their extent of heterozygote excess can be compared across age classes [43]. The observed differences of heterozygosity of the MHC and microsatellites in the different sexes and across age classes are thus more likely to reflect survival advantage of MHC *DRB *heterozygous males than an effect of population dynamics. Similarly, in wild baboons (*Papio ursinus*) older individuals had higher MHC class II *DRB *heterozygosity than younger individuals, independent from microsatellite heterozygosity, and *DRB *heterozygosity was greater in males than in females [43]. In many ungulates, males expend a substantial part of their fat reserve in the autumn rut. Less energy available for allocation to immune defense [44,45] together with adverse effects of testosterone [46-49] is the most likely explanation for the higher susceptibility of reproductively active males to pathogens. In chamois, increased susceptibility to scabies in sexually active males, as indicated by lower survival of these males in populations affected by scabies (Figure 4), is thus likely linked to their exhaustive depletion of energy reserves during the early-winter rut when they defend mating territories and compete heavily for access to females [35]. Indeed, our data confirmed a significant negative energy balance in chamois during winter, evident via a large decrease in body mass.

Further our analysis revealed a previously unknown but important detail: reproductively active males reached their lowest body mass as early as February, about six weeks before females, whereas younger males had a seasonal body mass trajectory more similar to that of females. This is in line with the mortality data: reproductively active males were less likely to survive than same-aged females, a difference that was not found among younger individuals. This sex difference in mortality was more pronounced in populations that had been affected by scabies (see also Ref.[50]). While our data does not demonstrate a direct link between MHC variation and body condition, this correlation strongly suggests that towards the end of winter males rely on an efficient MHC-mediated immune response to combat scabies infection. A higher susceptibility of rutting males to scabies is also supported by the fact that male chamois generally carry a higher parasite burden [34,51].

When comparing the MHC class II *DRB *allele frequencies between chamois populations exposed to scabies with those in which scabies epidemics have never been reported, we found that the MHC alleles profoundly differed in frequency (Figure 2). Such a variable distribution is unlikely to be the result of geographical differences as previous population genetic work has shown that the overall nuclear genetic differentiation is rather low among chamois populations in the Eastern Alps [52]. Scabies epidemics usually occur in waves interrupted by periods of low prevalence [34]. In the province of Styria (Austria) the two most recent peaks of the epidemic occurred between 1966 and 1970 and 1983-1987, respectively [53]. In the province of South Tyrol (Italy) (see Figure 1), the epidemic has recently peaked again after the previous episode lasting from 1980-1985 [54]. Therefore, most of our samples for genotyping were obtained after the last significant waves had waned. However, the frequency of the most dominant allele (169) was about two times higher in the scabies-exposed populations, which may explain why heterozygosity at the MHC locus was lower in these areas. The high abundance of allele 169 in populations known to have been affected by scabies in the past, together with the evidence for selection at the MHC class II *DRB *locus [41], suggests that carriers of this allele were less susceptible to scabies. As a consequence, allele 169 is likely to have increased in frequency, mostly at the expense of allele 162. Furthermore, a previous study reported that chamois herds in an area of north-eastern Italy recovered quite fast after a severe scabies infection and two subsequent scabies epizootics had a less severe impact on the population [34], suggesting a superior immunogenetic status of chamois that survived the initial scabies outbreak. Interestingly, the oldest females (20 years of life) in our study were all homozygous carrying the allele 169. This allele, which corresponds to the putative amino acid sequence Ruru-DRB*01 (GenBank accession no: AAQ75528), was found to be one of the most frequent alleles in closely related Pyrenean chamois [40], a species also known to be affected by scabies [55]. The MHC allele 162, which corresponded to the putative amino acid sequence Ruru-DRB*18 (GenBank accession no: AAQ75545), has not been detected in the Pyrenean chamois or may not occur at all in that species. These two alleles differ at 5 amino acid positions, all of them are located specifically at antigen binding sites (amino acid positions 11S, 70Q, 71 T, 78Y) [38]. These specific amino acid positions in the exon 2 of that locus were also found to be under strong positive selection in various other ungulate species [41]. However, in our study we did not find any statistically significant association of MHC alleles and individual survival rate in scabies and non-scabies populations. Certainly, chamois are affected by a plethora of pathogens [45,51] and hence high MHC heterozygosity may result from the advantage of allelic variation to combat this range of simultaneously occurring pathogens. However, the evolutionary arms race between the host's immune defences and various parasite adaptation strategies may prevent fixation of specific MHC alleles. In Soay sheep (*Ovis aries*) a reported association between MHC variation, parasite resistance, and juvenile survival rate suggests that different MHC alleles may exhibit different associations with parasites at various stages during individual lifespan [56]. In fact, experimental infection of mice with multiple strains of *Salmonella *has shown that MHC heterozygotes have enhanced clearance rates of infections, but this benefit was due to resistance being dominant rather than overdominant [4]. We therefore suggest, based on our current data, that the heterozygosity affect in chamois may be due to dominance rather than overdominance at the MHC.

A potential confound of our study could be that the higher mortality detected for reproductively active males compared to females and younger males results from hunting practices (if breeding males are the preferred game). However, hunting is unlikely to have been selective with respect to MHC genotype and hunting practices most likely do not profoundly differ in the Eastern Alps in populations affected by scabies or not. In addition, longevity of males older than four years from the Eastern Alps populations where scabies had never occurred was not different from females and similar to that reported for the Maritime Alps Regional Park (Western Alps) population [29]. Therefore, impaired survival of reproductively active males in scabies affected areas is better explained by the higher susceptibility of individuals in poor body condition at the end of the winter season and potentially in combination with other factors such as MHC variability.

## Conclusion

The survival advantage for chamois males heterozygous at the MHC class II *DRB *locus suggests a fitness benefit in terms of parasite resistance (e.g., scabies), particularly for reproductively active males with depleted energy reserves due to rutting behaviour. As a consequence, MHC heterozygous males live longer than homozygotes. This scenario renders the MHC-mediated immune response more important for males than for females, which implies a relatively stronger selection pressure on MHC genes in males than in females. Similar scenarios are likely to exist in other species. Therefore, considering sex-specific effects of MHC alleles may help to clarify the often inconsistent empirical evidence about the adaptive value of MHC variability.

## Methods

### Samples and study regions

From 1997 to 2009 we obtained liver tissue for genetic analyses from 190 females and 174 males harvested during regular hunting throughout the Eastern Alps in Austria and the province of South Tyrol, Italy (geographic locations of the 22 chamois sampling sites are given in Figure 1; see also for details in Additional file 2: Table S2). For the analysis of survival and seasonal changes of body mass and condition, we used additional data, obtained between 1970 and 1981 from 1001 individuals (497 males and 504 females) harvested by professional game wardens in the Eastern Alps mountain area of Achenkirch (Austria) (Figure 1). All animals were taken during hunts for population management purposes by licensed managing authorities, and were not killed specifically for this study. From all 1365 sampled chamois in the present study (671 males and 694 females) we knew sex and age at death determined by external inspection and counts of visible annual horn growth layers [57]; for a subsample (532 males and 515 females) of the total samples, we also knew eviscerated body mass (details in Additional file 3: Table S3); for 497 males and 504 females of this subsample we further knew visceral and kidney fat mass. Information on recorded scabies outbreaks in each chamois population was provided by veterinary authorities, and derived from literature [33,35,53]. The geographic locations of populations that were affected by scabies epidemics and populations in which scabies epidemics has never been reported, are presented in Figure 1 and in Additional file 2: Table S2.

### DNA isolation and MHC genotyping

Liver samples were preserved frozen at -20°C and genomic DNA was extracted with the DNA extraction kit DNeasy Tissue Kit (Qiagen GmbH, Hilden, Germany) according to the manufacturer's protocol and stored in 200 μl distilled water. We used capillary electrophoresis single-strand conformation polymorphism (CE-SSCP) to screen the allelic (i.e. exon 2) diversity. This method can be automated and is characterized by high throughput, high sensitivity and good reproducibility [58-60]. The exon 2 was amplified using the fluorescent labeled primers HL030 (6'-FAM - forward strand) and HL032 (NED - reverse strand) [38] and the resulting amplicon-size was 284 bp including the primers. For all PCRs, the Qiagen Multiplex PCR Kit (Qiagen GmbH, Hilden, Germany) was used. The thermal cycling profile for the PCRs consisted of initial heating at 95°C for 15 min (hot-start polymerase activation), followed by 30 cycles of denaturation at 94°C for 30 sec, annealing at 56°C for 30 sec, extension at 72°C for 90 sec, and ending with a 10 min extension step at 72°C. For the CE-SSCP analyses, the fluorescent-labelled PCR samples were prepared for electrophoresis by combining 1 μl PCR product with 14 μl loading mix which consisted of 13.75 μl Hi-DI formamide and 0.25 μl Genescan ROX 350 standard (Applied Biosystem). The mixture was heated for 3 min at 95°C to separate the complementary DNA strands, chilled on ice for 4 min and analyzed by capillary electrophoresis on an ABI PRISM^® ^3100 automated DNA Sequencer (Applied Biosystem). The CE-SSCP polymer consisted of 5% Genescan polymer (Applied Biosystem), 10% glycerol, 1xTBE, and HPLC-water. The running buffer mixture contained 10% glycerol, 1xTBE and HPLC-water. The separation of the allelic variants was achieved by run conditions at 12 kV for 36 minutes and by a run temperature at 22°C. The retention times of the sequence variants were identified relative to the ROX 350 standard. The GeneMapper software package 4.05 from Applied Biosystems was used to process the obtained SSCP data. All samples with a single CE-SSCP peak (i.e. putative homozygous individuals) were also subjected to direct sequencing to confirm or discount homozygosity. Moreover, previously cloned and sequenced samples [38] were used as reference samples to assign CE-SSCP peaks to the corresponding nucleotide sequences. Both DNA strands were sequenced using the HL030 and HL032 primers as sequencing primers and the Applied Biosystems BigDye Terminator v3.1 Cycle Sequencing Kit. Sequencing and data processing was carried out on the ABIPRISM 3100 automated DNA Sequencer (Applied Biosystem). Sequence analysis was carried out using the software Bioedit version 7.0.9 (http://www.mbio.ncsu.edu/BioEdit/bioedit.html). The obtained MHC class II *DRB *sequences in this study are available on GenBank (accession numbers: AY368437, AY368440, AY368441, AY368443-AY368446, AY368449, AY368451-AY368455, EU887495, EU887500, and EU887506; see also Additional file 1: Table S1).

### Microsatellite genotyping

For the microsatellite analysis we used chamois suitable loci that were already tested and used in other studies [61,62]. Each sample was at following 12 microsatellites genotyped (annealing temperatures in parentheses, if deviating from the author's reference): RMO26 and RMO29 [61,63] INRA005, INRA011 (52°), INRA023, INRA036 (52°), ETH10 (55°), ETH225 (52°), SR-CRSP01 (52°), SR-CRSP05 (52°), SR-CRSP08, SR-CRSP09 (50°) [62]. The PCR products were electrophoresed on a LI-COR 4200 automated sequencer along with a fluorescently labelled size standard (50-350 bp sizing standard; LI-COR^® ^Biotechnology Division). Allele lengths were determined using Gene ImageIR ver. 3.52.software (LI-COR, Inc., ^© ^1990-1998).

### Data analyses

In a previous population genetic study, we have shown that the overall nuclear genetic differentiation is rather low among chamois populations in the Eastern Alps [52]. In the present study, we used the Arlequin program (version 3.5.1.2) [64] to calculate mean gene diversity (expected heterozygosity (H_E_)) and *F*_ST_-values for chamois populations in which scabies has never been reported and for populations that have been exposed to scabies epidemics. Individual heterozygosity for microsatellite loci was calculated as the number of heterozygous microsatellite loci divided by the number of loci (i.e., 12 loci) examined.

Statistical analyses were performed using the package R [65] with *P*-values derived from two tailed tests. Except when otherwise stated, data were analyzed by linear modeling, or, in the case of binomial response variables, by generalized linear modeling. Justification of parametric testing was checked with diagnostic tools available in R and visual inspection of residuals. We followed the principal of model simplification by removing terms to achieve a model with the smallest value for Akaike's Information Criterion (AICc, AIC corrected for small sample size), which weighs the goodness of fit of competing models against by the number of terms included. To find this model we used an exhaustive computation of all possible models (R package "MuMIn" [66]). For identifying the relative importance of predictor variables and to obtain unconditional coefficients and standard errors, we used model averaging based on AICc. From all possible models we considered only those with a Δ AICc < 4 compared with the model with the lowest AICc.

For analysing survival, we calculated Cox's proportional hazard models (procedure 'coxph', R-package 'Survival' [67]). Since we used the age structure of the hunted chamois, or that of carcasses found, respectively, the age specific hazard was estimated solely from uncensored cases. Significance of seasonal variation of body mass was tested by entering a sine (t) and cosine (t) term into regression models, with t representing month of year in radians. Sums of squares and degrees of freedom of these terms were added to obtain a single *F *and *P *value for the periodic function. Coefficients of the sine and cosine terms were then used to algebraically compute the amplitude (A) and phase (ϕ) of the periodic function. Confidence intervals for A and ϕ were determined by least-square estimates of these parameters of a non-linear model. In addition, we tested whether the age structure in our sample of hunted chamois reflected natural mortality by comparing it with 508 male and 359 female age-known carcasses found during a study in the Western Alps, the Maritime Alps Regional Park in Italy [29].

## Authors' contributions

SH & SF conceived study; SH, BSH and SS performed laboratory work; SF& AW organized tissue and data sampling, SH, MDL and AW analyzed data; SH, AW & FS wrote the paper. All authors read and approved the final manuscript.

## Supplementary Material

Additional file 1 **Table S1**. MHC class II *DRB *alleles indentified by CE-SSCP and the corresponding nucleotide sequences. Click here for file

Additional file 2 **Table S2**. Sampling sites affected by scabies epidemics and areas in which scabies epidemics were never recorded. Click here for file

Additional file 3 **Table S3**. Sample sizes of body mass data of genotyped and not genotyped individuals at the different sampling sites. Click here for file
