# Supplementary material for: Sex-specific selection for MHC variability in Alpine chamois
Source: BMC Evol Biol. 2012 Feb 15;12:20. doi: 10.1186/1471-2148-12-20 (PMC3340304; doi:10.1186/1471-2148-12-20)
Supplement: Additional file 1 — Table S1. MHC class II DRB alleles indentified by CE-SSCP and the corresponding nucleotide sequences. [file 1471-2148-12-20-S1.DOCX]

**Additional material**

**Additional file 1: Table S1:** MHC class II *DRB* alleles indentified by CE-SSCP and the corresponding nucleotide sequences and the corresponding GenBank accession numbers.

| **CE-SSCP**  **MHC-*DRB* allele** | **Nucleotide sequence**  **MHC-*DRB* allele** | **GenBank**  **accession numbers** |
| --- | --- | --- |
| 161 | Ruru-DRB*17 | AY368453 |
| 162 | Ruru-DRB*18 | AY368454 |
| 163 | Ruru-DRB*09 | AY368445 |
| 164 | Ruru-DRB*10 | AY368446 |
| 165 | Ruru-DRB*31 | EU887500 |
| 166 | Ruru-DRB*04 | AY368440 |
| 167 | Ruru-DRB*13 | AY368449 |
| 168 | Ruru-DRB*19 | AY368455 |
| 169 | Ruru-DRB*01 | AY368437 |
| 170 | Ruru-DRB*05 | AY368441 |
| 171 | Ruru-DRB*26 | EU887495 |
| 172 | Ruru-DRB*35 | EU887506 |
| 174 | Ruru-DRB*16 | AY368452 |
| 175 | Ruru-DRB*08 | AY368444 |
| 176 | Ruru-DRB*15 | AY368451 |
| 179 | Ruru-DRB*07 | AY368443 |
