# Supplementary material for: Sex-specific selection for MHC variability in Alpine chamois
Source: BMC Evol Biol. 2012 Feb 15;12:20. doi: 10.1186/1471-2148-12-20 (PMC3340304; doi:10.1186/1471-2148-12-20)
Supplement: Additional file 2 — Table S2. Sampling sites affected by scabies epidemics and areas in which scabies epidemics were never recorded. [file 1471-2148-12-20-S2.DOCX]

**Additional file 2: Table S2:** Number of genotyped chamois samples of different sampling sites from the Eastern Alps (Austria and Italy), affected by scabies epidemics and areas in which scabies epidemics were never recorded. Given is also age range of sampled individuals at each sample site.

| **Sampling site** | **Acronym  see Fig. 1** | **Scabies *vs*. non-scabies** | **Sample size** | **Age range (median)** |
| --- | --- | --- | --- | --- |
| District Brenner (Italy) | BRE | scabies | 77 | 1 – 15 (6) |
| Corvara (Italy) | COV | scabies | 47 | 0 – 17 (4) |
| Gailtaler Alpen | GAI | scabies | 10 | 1 – 16 (4.5) |
| Hagengebirge | HAG | scabies | 13 | 0 – 13 (7) |
| Hundstein | HUN | scabies | 4 | 0 – 5 (3.5) |
| Kalkalpen | KAL | scabies | 16 | 0 – 12 (2) |
| Karnische Alpen | KAN | scabies | 17 | 0 – 20 (3) |
| Karawanken | KAW | scabies | 4 | 0 – 30 (2.5) |
| Kitzbuehler Alpen | KIT | scabies | 4 | 0 – 10 (1.5) |
| Kreuzeckgruppe | KZK | scabies | 14 | 1 – 12 (4) |
| Nockberge | NOB | scabies | 21 | 0 – 15 (4) |
| Osterhorngruppe | OHG | scabies | 6 | 1 – 8 (4) |
| Schladminger Tauern | SCH | scabies | 19 | 0 – 14 (7) |
| Hohe Tauern | TAU | scabies | 49 | 0 – 15 (5) |
| Tennengebirge | TEN | scabies | 11 | 0 – 20 (8) |
| Achenkirch | ACH | non-scabies | 13 | 0 – 13 (3) |
| District Bruck an der Mur | BAM | non-scabies | 4 | 6 – 9 (7) |
| Koralm | KOR | non-scabies | 3 | 0 – 6 (2) |
| Rax | RAX | non-scabies | 2 | 2 – 9 (5.5) |
| Saualm | SAU | non-scabies | 3 | 1 – 6 (5) |
| District Scheibbs | SBS | non-scabies | 23 | 0 – 19 (7) |
| Texel (Italy) | TEX | non-scabies | 4 | 8 – 12 (10) |
