# Supplementary material for: Sex-specific selection for MHC variability in Alpine chamois
Source: BMC Evol Biol. 2012 Feb 15;12:20. doi: 10.1186/1471-2148-12-20 (PMC3340304; doi:10.1186/1471-2148-12-20)
Supplement: Additional file 3 — Table S3. Sample sizes of body mass data of genotyped and not genotyped individuals at the different sampling sites. [file 1471-2148-12-20-S3.DOCX]

**Additional file 3: Table S3:** Availability of body mass data from genotyped and not genotyped chamois of different sampling sites from the Eastern Alps (Austria and Italy).

|  |  | **Body mass sample size** | | | | | | |  |
| --- | --- | --- | --- | --- | --- | --- | --- | --- | --- |
| **Sampling site** | **Acronym  see Fig. 1** | **Genotyped** | | | **Not genotyped** | | | |  |
|  |  | **♂<5y** | **♂>4y** | **♀** | | **♂<5y** | **♂>4y** | **♀** | |
| District Brenner (Italy) | BRE | 26 | 3 | 47 | | 0 | 0 | 0 | |
| Corvara (Italy) | COV | 2 | 7 | 15 | | 0 | 0 | 0 | |
| Gailtaler Alpen | GAI | 0 | 0 | 0 | | 0 | 0 | 0 | |
| Hagengebirge | HAG | 0 | 0 | 0 | | 0 | 0 | 0 | |
| Hundstein | HUN | 0 | 0 | 0 | | 0 | 0 | 0 | |
| Kalkalpen | KAL | 2 | 6 | 7 | | 0 | 0 | 0 | |
| Karnische Alpen | KAN | 0 | 0 | 0 | | 0 | 0 | 0 | |
| Karawanken | KAW | 0 | 0 | 0 | | 0 | 0 | 0 | |
| Kitzbuehler Alpen | KIT | 0 | 0 | 0 | | 0 | 0 | 0 | |
| Kreuzeckgruppe | KZK | 3 | 0 | 3 | | 0 | 0 | 0 | |
| Nockberge | NOB | 1 | 3 | 2 | | 0 | 0 | 0 | |
| Osterhorngruppe | OHG | 0 | 0 | 0 | | 0 | 0 | 0 | |
| Schladminger Tauern | SCH | 0 | 0 | 0 | | 0 | 0 | 0 | |
| Hohe Tauern | TAU | 0 | 0 | 0 | | 0 | 0 | 0 | |
| Tennengebirge | TEN | 0 | 0 | 0 | | 0 | 0 | 0 | |
| Achenkirch | ACH | 2 | 6 | 5 | | 130 | 331 | 441 | |
| District Bruck an der Mur | BAM | 1 | 0 | 1 | | 0 | 0 | 0 | |
| Koralm | KOR | 0 | 1 | 0 | | 0 | 0 | 0 | |
| Rax | RAX | 0 | 0 | 0 | | 0 | 0 | 0 | |
| Saualm | SAU | 0 | 0 | 2 | | 0 | 0 | 0 | |
| District Scheibbs | SBS | 6 | 5 | 10 | | 0 | 0 | 0 | |
| Texel (Italy) | TEX | 0 | 0 | 0 | | 0 | 0 | 0 | |
